# BARIATRIC DIET GUIDE: PLATE MODEL TEMPLATE FOR BARIATRIC SURGERY PATIENTS

**DOI:** 10.1590/0102-672020180001e1375

**Published:** 2018-07-02

**Authors:** Maria Paula Carlini CAMBI, Giorgio Alfredo Pedroso BARETTA

**Affiliations:** 1 Clínica Baretta, Curitiba, PR, Brazil

**Keywords:** Food guide, Plate model, Bariatric surgery., Guia alimentar, Modelo de prato, Cirurgia bariátrica.

## Abstract

***Background*:**

The Bariatric Plate Model (BPM) may be an adequate form of nutritional guideline after obesity surgery.

***Aim*:**

Create a food guide, based on the Plate Model for nutritional education of bariatric patients.

***Method*:**

The Plate Model^2^ was revised from a model initially suggested for dyslipidemic and hypertensive patients to a new objective: adaptation to bariatric patient who needs effective long-term nutritional education.

***Results*:**

The adaptation of the Plate Model considered protein needs with high biological value, as it is the priority in the BPM, followed by vitamins and minerals and lastly the carbohydrates, especially the whole ones.

***Conclusion*:**

The BPM is a tool that can be effectively used in nutritional education of bariatric patients.

## RESUMO

***Racional*:**

o Modelo de Prato Bariátrico (MPB) pode ser uma forma adequada de orientação nutricional após a cirurgia da obesidade.

***Objetivo*:**

Criar um guia alimentar, baseado no Modelo de Prato para educação nutricional de pacientes bariátricos.

***Método*:**

Foi revisado o The Plate Model^2^, modelo de prato sugerido inicialmente para pacientes dislipidêmicos e hipertensos com o intuito de adaptá-lo ao paciente bariátrico que necessita de educação nutricional efetiva em longo prazo.

***Resultados*:**

A adaptação foi feita considerando que o uso de proteínas de alto valor biológico é a prioridade no MPB, seguido de vitaminas e minerais e por último os carboidratos, especialmente os integrais.

***Conclusão*:**

O MPB é ferramenta que pode ser usada de maneira efetiva na educação nutricional de pacientes bariátricos.

## INTRODUCTION

The expansion of epidemic obesity and surgical treatment to reduce its impact on the health brings a new perspective on the way to treat and maintain both weight and nutritional status of those who underwent the surgery. The surgical process is effective in combating obesity grades II and III, and, due to this, there is an exponential increase in the number of operations performed around the world^5^.

The patient undergoing bariatric surgery, whether by means of mixed gastric bypass technique or by restrictive vertical gastrectomy, must maintain a strict nutritional order for promoting subcutaneous fat loss and preservation of muscle mass^5^. The operation per se promotes the desired weight loss, but food reeducation and physical activity are priorities. The most commonly used food guide is the Food Pyramid^13^, but it does not accurately translate to the patient how to compose the daily meals. 

The process of food re-education is fundamental for many types of situations that require special nutritional care. The first plate model came up to treat cardiopath and dyslipidemic patients^2^. It is a simple form of nutritional guidance because it aims at the greater goal of the patients under orientation, which is their understanding in the daily reality in which they live. Facilitating the educational process is the role of the nutritionist. For orientation, it is necessary to create a clear and practicable method for the person undergoing a surgery. 

With this objective, this study seeks to discuss the Bariatric Plate Model (BPM), which is a simple way to demonstrate to the patients how the macro and micronutrients can be distributed in their daily meals, so as to favor their weight loss and the maintenance of their nutritional status in the long term.

## METHOD

The Plate Model^2^ was revised; it was a model of plate suggested initially for dyslipidemic and hypertensive patients. Adaptation made to the bariatric patient, who needs effective long-term nutritional education, to orientate the consumption of adequate amounts of protein with high biological value, followed by vitamins and minerals and lastly the carbohydrates, especially the whole ones 

## RESULTS

Bariatric surgery is an effective form of weight loss; however, for long-term results, food quality should be prioritized, as the volumetric capacity of the stomach is reduced and requires supplementary nutritious foods. There is a natural change in the alimentary profile of the patient by reducing the intake of sweets in general, which are highly palatable and energetic, and an increase in the consumption of hyperproteic foods^8^.

The everyday life of the patients should be simplified with a more easily understandable way of composing their daily meals. Therefore, it is necessary to demonstrate, through the composition of the BPM, how to plan the plate from the first meal to the last of the day, and how important the nutrients are in the choices they will make.

### Caloric restriction

Bariatric surgery, both by restrictive and mixed technique, requires a reduction in daily caloric intake compatible with the reduction of gastric pouch. Thus the daily calorific value ingested by a patient starts on average with 500 kcal in the liquid feed and progressively evolves to a solid consistency up to 1.200 kcal per day. Nutritional recommendations following bariatric surgery are described in guidelines that mention the need for protein from 1-1.5 g/kg of ideal weight (60-80 g/day, 25%), carbohydrates (45%) and lipids (30%)^1^.

Macro and micronutrients are very important for the maintenance of the health of the patient. Among the macronutrients, the most important one is the protein.

### Proteins

Proteins are biological macromolecules made up of one or more chains of amino acids and participate in almost all cellular processes. They have essential functions such as DNA replication, molecular transport and response to stimuli. They function as enzymes to catalyze chemical reactions vital to metabolism, participate in the cell cycle and in the immune response. Proteins differ fundamentally in their amino acid sequence, which is determined by the genetic sequence and which generally causes their knotting in a specific three-dimensional structure that determines their activity^1^.

The essential amino acids are those which the body is not able to synthesize by itself and must be obtained by the consumption of foods that contain proteins, which are transformed into amino acids during the digestion^1^.

Protein sources can be found in a wide variety of plant and animal foods. Meat, eggs, milk and fish are complete sources. Among the main vegetables rich in protein are the vegetables, especially beans, lentils, soybeans and chickpeas. Most amino acids are available in human food, but special situations, such as bariatric surgery, require supplementation. When the body does not receive the necessary amounts of protein, there is protein insufficiency and malnutrition, which can lead to a number of diseases, including kwashiorkor, alopecia and intense muscular loss^1^.

Many patients may develop intolerance to iron-rich protein foods due to inadequate mastication and also by the decrease of hydrochloric acid and proteolytic enzymes such as pepsinogen. Their consumption should be encouraged through specific chewing and portioning training sessions^11^.

Half (50%) of the plate must contain proteins.

For meals such as lunch and dinner, one should place sources of iron-rich proteins such as meats - beef, chicken, fish and eggs - to make up half of the plate, that is, 50% of the total to be ingested. Always use low fat options. The average food intake of the operated patients is around 4-6 tablespoons of food per meal. Therefore, it would be 2-3 tablespoons of food coming from proteins. As they are accompanied by some lipid content, the orientation is to use them in the baked, cooked or grilled form to minimize caloric value and facilitate consumption by the operated patient^11^.

For the breakfast or snacks, it is necessary to prioritize sources of calcium rich proteins, such as milk and dairy products. Start the day with skim milk, cottage-type cheeses, ricotta or Minas cheese (white cheese) and sugar-free yogurts. The use of yogurts is excellent for maintaining the consumption of natural probiotics, responsible for the rebalancing of intestinal bacteria and protection against intestinal dysbiosis^1^ ^,^ ^11^.

Iron-rich protein food sources should be used in separate meals such as lunch and dinner, calcium-rich meals such as breakfast, and snacks to promote the absorption of these micronutrients^12^ ^,2^.

Protein supplementation is critical. To achieve daily nutritional needs after bariatric surgery, the use of Whey Protein should occur throughout life. The use of powdered supplements should start as early as the first day of liquid feeding and be kept throughout life. It is ideal to use isolated, hydrolyzed, lactose-free, gluten-free and sucrose-free formula to facilitate use adhesion. The powder can be diluted in water by being better absorbed, or in skimmed milk^11^.

### Vitamins and minerals

One third of the plate (30%) should be occupied by the group of vitamins, minerals and fibers, represented by fruits and vegetables in general. They are fibrous foods that require chewing. It is important to vary the colors from day to day to strengthen the immune system, regenerate the skin and regulate metabolism. Moderation is the watchword. Excess vitamins and minerals can be dangerous. Some vitamins, such as sun exposure D, pyridoxine (B6) and biotin, are released by intestinal bacteria^11^.

The most important and most discussed nutrients are vitamins A, D, B_12_, B_1_, calcium and iron^1^ ^,^ ^11^.

The vitamins can be water soluble and fat soluble. In the BPM it is necessary that both be present. Water-soluble vitamins, such as those from complex B, need to be eaten raw to maintain their nutritional value^2^.

Using colors is ideal for motivating the consumption of the maximum of nutrients possible in the meals. The yellow and red ones are rich in vitamin A (fat soluble) and responsible for keeping hair, skin and nails healthy; their best sources are carrots, beets, pumpkin and bovine liver. The greens are rich in vitamins of the B complex, and represented by the leafy ones like cabbage, mustard, chard, lettuce and arugula, which prevent anemia. Citrus fruits are rich in vitamin C and important in iron fixation and enhancement of immunity; they are present in orange, lemon, passion fruit, acerola, green apple, tomatoes and grapes. The white ones, such as onions, garlic, mushrooms, cauliflower, palm hearts, okra, are excellent in preventing cardiovascular disease and cancer^2^.

The vitamin B1 (tiamine) is present in the germ of wheat, beans, nuts, seeds and brown rice. It is important for the patients because it protects them from bariatric beriberi, which is a postoperative complication that can lead the patient to severe cardiological and neurological complications, edema and nutritional amblyopia. Its supplementation in severe situations can reach up to 100 mg/day^1^.

A complete multivitamin complex can meet the daily needs of vitamins, always coupled with sufficient food intake. Vitamin B2 or riboflavin is found in avocados, dairy products, eggs, nuts, wheat germ and yeast. It is very important in the cellular respiration, maintenance and restoration of the tissues. Vitamin B3 or niacin is essential for skin health, participates in the metabolism of carbohydrates, protects the digestive system and nervous system. Its richest sources are fish, liver, meat, yeast, peanut and whole grains. Vitamin B5 or pantothenic acid is important for macronutrient metabolism and maintenance of the nervous system. It is also produced by intestinal bacteria. Vitamin B6 or pyridoxine participates in the metabolism of proteins and in the formation of red blood cells found in bananas, fish, potatoes and also produced by intestinal bacteria. Vitamin B9 or folic acid present in dark green leaves and orange participates in DNA production, cell division, neural tube formation of the fetus, formation of red and white blood cells and protection against pernicious anemia, common in bariatric patients^1^. The vitamin B12 or cyanocobalamin, on the contrary, is present only in foods of animal origin, normally rich in proteins such as meats, milk and their derivatives. Its absorption is greatly impaired after gastric bypass due to the reduction of the intrinsic factor of the stomach and loss of its absorptive site, which is the ileum. Its lack is risky to the nervous system, because it can cause forgetfulness, irritability, difficulty of concentration and tingling in hands and feet. Even if the patient consumes these foods, this vitamin must be permanently replenished throughout life, either orally, intramuscularly or sublingually^1^ ^,^ ^11^. Biotin (vitamin H) is from the B-complex and is not dosed by traditional hematological tests, but it is very important for maintaining the health of hair, skin and nails and should be restored whenever there is a complaint. In usual food it is present in egg yolk, wheat germ and also produced by intestinal bacteria^14^.Vitamin D or cholecalciferol is important for weight maintenance and also for bone metabolism. Their dietary sources are limited to milk and dairy products, eggs and liver. Its biggest source is sunlight, so it is recommended that the patient sunbathe daily, preferably without sunscreen for 15 min. Synthetic supplementation is routine in both the previous and postoperative period, on average 2000 UI per day^6^. Vitamin E (fat soluble vitamin) or tocopherol is a potent antioxidant and protective of cell membranes. Its best sources are almonds and milk. Vitamin K (fat soluble) - menaquinone or phylloquinone - is important for blood clotting and produced by intestinal bacteria. The best sources are broccoli, cabbage and kale^1^ ^,^ ^11^.

The food sources of vitamins and minerals in general are confused with other groups of macronutrients, as is the case of the vitamin B12 and zinc, which have as their main sources the foods rich in animal proteins such as meats, chicken, milk and their derivatives.

### Carbohydrates

The rest of the plate should consist of carbohydrates, which are energetic foods, important for daily life. The choice in this group is for the whole-grain foods. Whole carbohydrates with breads, rice, pasta and cereals tend to decrease the absorption of sugars and fats, which favors cardiovascular health, in addition to promoting better satiety power^15^.

**FIGURE 1:**
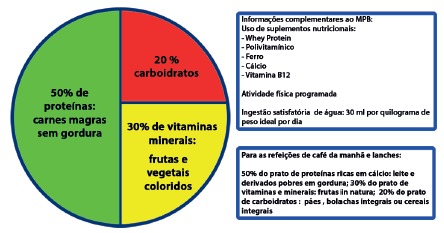
Diagrammatic composition of a bariatric plate (BPM) and associated activities

### Lipids

They are insoluble chemicals in water. They are important macronutrients to provide essential fatty acids. Suggested sources are: canola oil and olive oil. Canola oil is encouraged because it is safe for humans, as well as for its positive effects on variables such as reduced tumor cell growth, increased antioxidant capacity, increased insulin sensitivity and glucose tolerance as well as reduction of total triacylglycerol and LDL cholesterol^9^. The prevention of alopecia in operated patients must also be mentioned. Olive oil, which is a common lipid source in the Mediterranean diet, rich in oleic acid, a monounsaturated fatty acid (ω9) that is present in concentrations higher than 50% in olive oil ^2^.

### Nutrition supplements

The use of nutritional supplements is mandatory and requires periodic metabolic control to analyze the need for each specific nutrient. The use of Whey Protein can improve body composition in women and also prevent weight return^11^ ^,^ ^10^.

The ideal amount of proteins is up to 30 g per meal in the first year after the operation. As the food intake is lower than expected, we encourage the use of Whey Protein, one to two scoops per day, with an average of 25 g of protein as measure^10^; vitamin B12 in the monthly intramuscular dose of 5000 mcg or 350 mcg orally per day; iron in 18 mg orally for men, 50-100 mg orally for women in childbearing age. In some specific cases there may be a need to use intravenous iron (ferritin below 30 mg/dl); calcium with 2000 mg per day; enough polyvitamin to reach 200% of the RDA for micronutrients^1^ ^,^ ^7^.

### Water consumption

Water is a fuel for various reactions in the body and plays a key role in intestinal, cerebral, pulmonary, renal and cardiological functioning. With a consumption of 30 ml/kg of ideal weight per day it is possible to avoid the formation of gallstones and renal calculus^11^.

### Physical activity

The practice of daily physical activity is encouraged from the 30^th^ day after bariatric surgery. The physical educator should plan and guide the proper exercise for each patient. The major goal should be the preservation and recovery of lean mass and elimination of fat mass. With this, there is greater chance of long-term weight maintenance^4^.

## CONCLUSION

The Bariatric Plate Model may be a good form of nutritional education, also highlighting the protein intake as a macronutrient basis. Together with this, the water intake, the use of supplements and the physical activity must be incorporated in the routine of the patient.
